# Molecular symmetry change of perfluoro-n-alkanes in ‘Phase I’ monitored by infrared spectroscopy

**DOI:** 10.1007/s44211-024-00611-w

**Published:** 2024-06-14

**Authors:** Taisuke Araki, Takayuki Oka, Nobutaka Shioya, Takeshi Hasegawa

**Affiliations:** Laboratory of Chemistry for Functionalized Surfaces, Division of Environmental Chemistry, Institute for Chemical Research, Kyoto University, Gokasho, Uji, Kyoto 611-0011 Japan

**Keywords:** PFAS, Phase diagram, Perfluoroalkyl compounds, IR spectroscopy, Raman spectroscopy, Band progression

## Abstract

Phase diagram of polytetrafluoroethylene (PTFE) comprises four regions. Phases II and IV are characterized by twisted perfluoroalkyl (R_f_) chains having different twisting rate of 13/6 and 15/7, respectively, while Phase III is characterized by a planer trans-zigzag molecular skeleton like a normal alkyl chain. These are confirmed by X-ray and electron diffraction and have already been established. Unlike these, Phase I is left an unresolved matter. This phase is complicated indeed and is not symbolized by a single molecular structure. At an ambient pressure, Phase I is the temperature region above 30 ºC (303 K), and the helical molecular structure is supposed to be gradually untwisted with an elevating temperature. This untwisting image is roughly suggested by the diffraction, neutron scattering, and thermal expansion techniques, but the conventional approaches have all experimental limitations because the untwisting accompanies disorder (or defect) in the twist along the chain. To explore the transition between two different helical structures of the R_f_ chain having disordered structures, vibrational spectroscopic techniques are expected to be an alternative approach. For infrared spectroscopy, for example, the twisting rate of the molecule is simply recognized as a degree of molecular symmetry. Here, we show that the band progression peaks of the CF_2_ symmetric stretching vibration mode are quite sensitive and useful for pursuing the molecular symmetry change in Phase I for both peak intensity and position using perfluoro-n-alkanes having different chain length covering both even and odd number of the CF_2_ groups.

**Graphical abstract:**

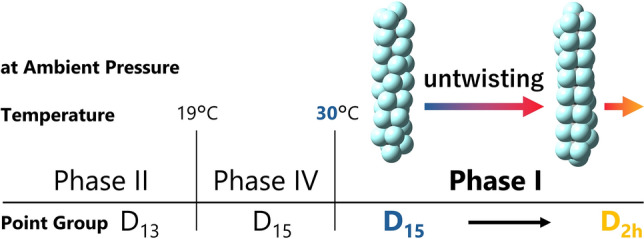

**Supplementary Information:**

The online version contains supplementary material available at 10.1007/s44211-024-00611-w.

## Introduction:

Poly- and perfluoroalkyl substances (PFAS) that involves a chemical moiety of a perfluoroalkyl (R_f_) group with various chain lengths [1, 2] have long been used for huge variety of purposes because of their unique material properties that cannot be regenerated by non-fluorine compounds [3, 4]. One of the most shared properties is the water-and-oil repellence that is conveniently used for daily life, medical purposes, and industrial processes represented by the semiconductor manufacturing process. Nafion^®^ membrane [5–9] used as the key device for fuel cells is another important application taking advantage of a unique property of a highly branched PFAS membrane that allows protons and molecular water to pass through the membrane, but bulky water cannot [5–8, 10, 11]. This essential use of PFAS is a result of human wisdom with great efforts mostly paid in organic chemistry, i.e., molecular design, and synthesis followed by analysis of material characters.

Besides, PFAS have attracted skyrocketing attention in recent years because of global concerns about their environmental diffusion as well as bioaccumulation and toxicity in human and animals. PFAS are especially to blame for concerns of the extremely high chemical stability that has the other side of the characteristic that PFAS are never decomposed in nature. PFAS are indeed extraordinary compounds that the molecular mechanism for the unique material properties has been unclear in terms of molecular science for many years, and PFAS research has largely been oriented to development of new functionalized materials.

To address this situation, an entirely new chemical theory has recently been proposed to comprehensively explain how the material characteristics figure out on some fundamental physical parameters of (1) a large permanent dipole moment along the C–F bond, (2) a small atomic polarizability of fluorine, and (3) the helical conformation about the R_f_ group [11–13]. As a matter of fact, many researchers had ever kind of imagining that they are individually important for understanding of PFAS [3], but their correlation has never been theorized in a comprehensive manner. The new theory is on the stratified dipole-arrays (SDA) model [11, 13] that stands on the three physical factors that are cooperatively interrelated with each other, and the model reveals that molecular two-dimensional (2D) spontaneous aggregation happens with a perpendicular orientation in the aggregate if the chain length is C8 or longer. Once molecular aggregates of R_f_ groups are generated by local dipole–dipole interactions, the vector sum of the dipole 2D network becomes much smaller in absolute value on the macroscopic scale, and the macroscopic properties become quite different from those of single molecules.

This concept of molecular self-aggregation has an impact on the PFAS characteristics as well as the terminal groups such as carboxylic and sulfonyl groups. Thus far, most of the PFAS issues related to environment and human health have been discussed by classification of the terminal groups, chain length, and branching structure [1]. However, the intrinsic molecular property about the spontaneous aggregation has been missing, which is supported by a fact that the experimentally core factor of “molecular orientation” is not involved in the discussion [14] .

**Fig. 1:**
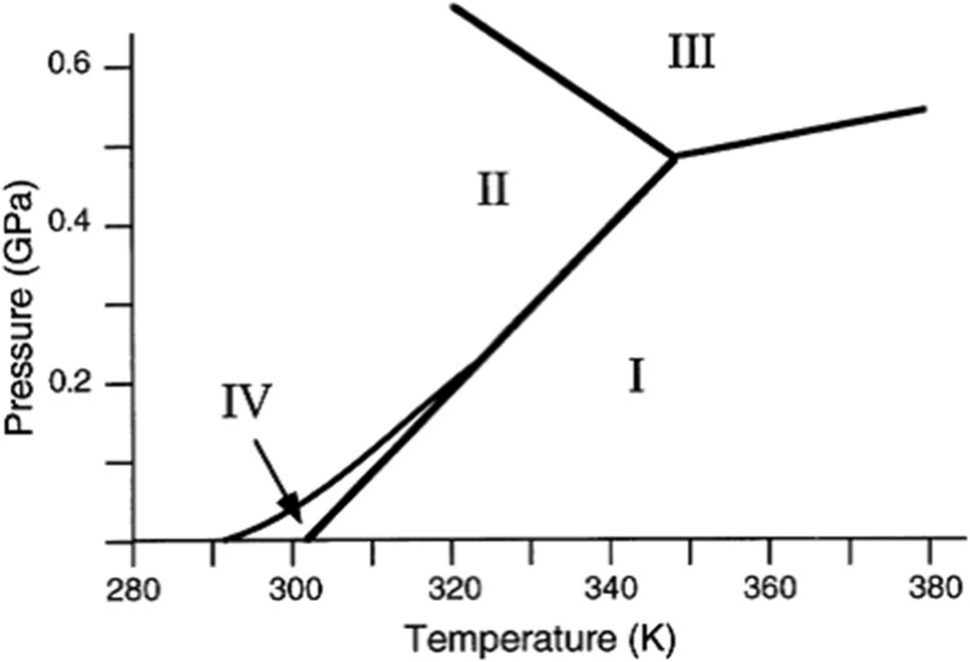
Phase diagram of temperature and pressure for PTFE [19] .

The helical structure [15–17] is a particularly important one among the three physical parameters to understand the molecular self-aggregation because it significantly depends on the temperature and pressure. Phase diagram of PFAS is established only for PTFE [17–22] as presented in Fig. 1. At the ambient pressure, we can focus on the abscissa axis only that has two transition temperatures of 19 and 30ºC (292 and 303 K, respectively) that are boundaries of Phase II, IV and I.

Each phase was mostly studied by using X-ray and electron diffraction and neutron scattering techniques, and Phase II and IV are found to have helical structures with a point group of *D*_13_ and *D*_15_ conformation [17, 19]. On the other hand, Phase I is exceptionally complicated that the helix is known to be untwisted from 15/7 helix toward the planer all-trans zigzag on elevating temperature [17, 23–26], which corresponds to the change of the point group of the molecular symmetry from *D*_15_ toward *D*_2h_, respectively [17, 23–26]. We have to note that, however, the untwisting accompanies disorder in the twist along the chain [17]. If the change would further be analyzed quantitatively, such a diffraction approach has a large experimental limit. In this situation, another experimental approach using vibrational spectroscopy is highly expected as employed for analysis of Phase III [27] .

In infrared (IR) and Raman spectra, in general, conformational changes appear as changes of molecular symmetry represented by the point group. In the case of hydrocarbons, a normal alkyl group takes *D*_2h_ in a crystallized matter, but it changes to take *C*_2v_ if the matter is melted making the molecule disordered completely [28]. In the case of R_f_ compounds; however, this kind of conformational change is not expected, since the CF_2_ groups are highly packed in narrow space to make the whole chain very stiff. As a result, very minor negligible conformational changes are found in each phase except Phase I.

Hsu and co-workers [29] conducted an interesting work on IR analysis of the conformational change using an artificially prepared untwisted perfluoro-n-alkanes (C8, C14, C16 and C20) by heating up to a state in Phase I. They prepared an evaporated thin film of the samples on a cold CsI surface at 7.5 K. This corresponds to fixation of heated sample in Phase I by flash freezing on the cold surface. Therefore, the as-spun film in an amorphous state at the low temperature consisted of nearly all-trans conformers reflecting Phase I. Since this is in a very unstable state, it changes its conformation to the stable one at about 180 K or higher in an irreversible manner. This change was readily pursued by developing the band progression (BP) peaks mainly related to the symmetric CF_2_ stretching vibration (ν_s_CF_2_) mode [30, 31].

Here, in our present study, we have performed conformational analysis of Phase I using perfluoro-n-alkanes with various chain lengths covering even and odd (C12–C16) studied using the BP peaks of IR spectra with an aid of Raman spectroscopy, in which the conformational change is found reversibly on changing temperature. We have found that the BP peaks appeared as the satellite peaks of the main absorption peak of the ν_s_CF_2_ band are quite sensitive and useful for studying the conformational change from *D*_15_ to *D*_2h_ in terms of both peak intensity and position.

## Experimental

A series of perfluoro-n-alkanes having different chain lengths used for our study are as follows. Perfluorododecane (n-C_12_F_26_; > 96%), octacosafluorotridecane (n-C_13_F_28_; > 96.0%), and dotriacontafluoropentadecane (n-C_15_F_32_; > 96.0%) were purchased from FUJIFILM Wako Chemical Corporation (Hiratsuka, Kanagawa, Japan). Perfluorotetradecane (n-C_14_F_30_; > 98%) and perfluorohexadecane (n-C_16_F_34_; > 98%) were purchased from Apollo Scientific Ltd (Stockport, Cheshire, UK). As a representative fluoropolymer, polytetrafluoroethylene (PTFE) in a powder condition was purchased from Sigma-Aldrich (St. Louis, MO, USA). The average particle size is 1 μm. All the samples are in solid state at ambient temperature, and they were used without further purification.

### In situ IR attenuated total reflection (ATR) spectra measurements

The temperature-dependent IR spectra of n-C_15_F_32_ in the heating and cooling processes were measured by the ATR technique using a PIKE Technologies (Madison, WI, USA) GradiATR™ single-bounce reflection attachment having an ATR prism of diamond equipped with a heater. The temperature was increased at the rate of 20 °C min^−1^ which was naturally cooled in an ambient air at 25 °C. The angle of incidence was fixed at 45°, and un-polarized light was used. After pressing the sample on the ATR prism, the in-situ IR ATR measurements were performed on a Thermo Fisher Scientific (Madison, WI, USA) Nicolet iS50 FT-IR spectrometer having deuterated triglycine sulfate (DTGS) and mercury cadmium telluride (MCT) detectors. For the IR measurements lower than 650 cm^−1^, the DTGS detector was used with a modulation frequency of 5 kHz. Other measurements than the lower wavenumber region, a highly sensitive MCT detector was used with a modulation frequency of 60 kHz. The accumulation was carried out 100 times for each spectrum, and the wavenumber resolution was set to 1 cm^−1^. The apodization function was set to Blackman-Harris throughout the study. All the measurements were performed under ambient pressure. While the heating measurements, the sample was found gradually volatized, which cannot be prevented completely, making the peak intensity influenced. Therefore, the IR spectra shown as follows are normalized using the peak intensity at 1155 cm^−1^. IR ATR spectra of solid samples of perfluoro-n-alkanes (C12–C16) and PTFE were measured by the same spectrometer and the same condition, but without using a heater.

### Raman spectra measurements

Raman spectra were measured using a Thermo Fischer Scientific (Madison, WI, USA) DXRxi imaging microscopic Raman spectrometer having a diode-pumped laser with an excitation wavelength of 532 nm, a grating having 900 lines mm^−1^, and an edge filter for eliminating the Rayleigh scattered light. The observable range of the Raman shift was 3000−50 cm^−1^. The bulk samples of perfluoro-n-alkanes (C12–C16) and PTFE in the solid state were individually put on a glass plate, and an objective 10 × lens with a confocal aperture with a pinhole of 25 μm width were used for both purposes of laser irradiation and collection of the Raman scattered light. The laser was irradiated at a constant power of 10.0 mW at the sample surface. The Raman spectrum was obtained with an exposure time of 4 s and with a total acquisition time of 150 s.

### In situ powder X-ray diffraction (XRD) measurements

Powder XRD measurements were performed on a Rigaku (Tokyo, Japan) SmartLab X-ray diffractometer equipped with an Anton Paar (Graz, Austria) DCS 500 domed sample stage and a CCU 100 temperature controller. The temperature was increased and decreased at the rate of < 20 °C min^−1^. Cu Kα radiation (*λ* = 0.15418 nm) was generated from a sealed-tube X-ray source operated at 50 kV and 40 mA. The parallel X-ray beam was used to ensure that the effects of uneven surfaces would be negligible. The beam was irradiated onto the surface of well-ground n-C_15_F_32_, and the scattered rays were detected by a Rigaku HyPix-3000 hybrid pixel counting detector at scattering angles, 2*θ*, ranging from 2° to 25°. The scanning speed was set to 2° min^−1^ with steps of 0.02°.

## Results and discussion

### Assignment of satellite peaks

**Fig. 2:**
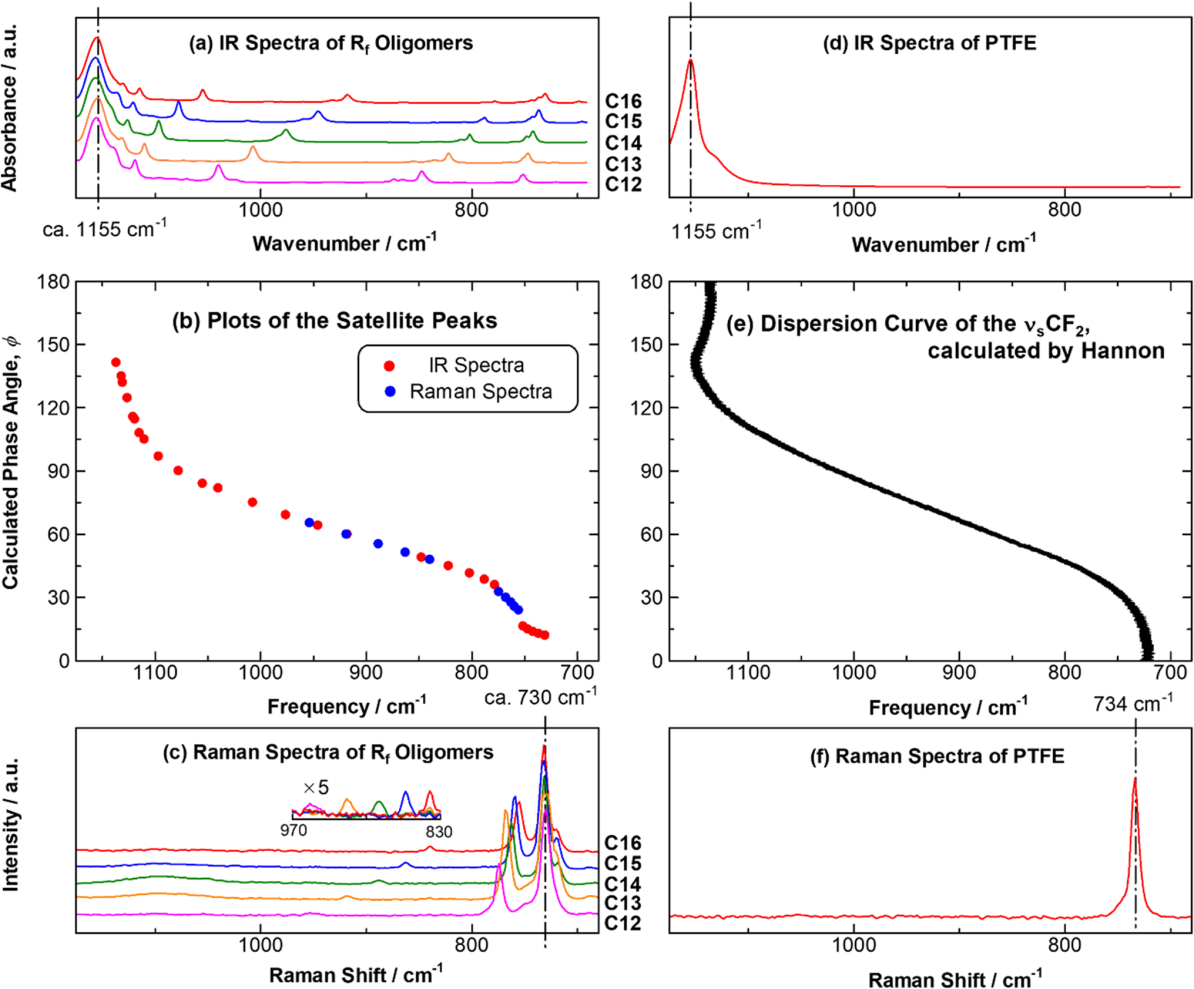
**a** IR spectra of perfluoro-n-alkanes with a length of C12–C16, **b** the plot of the satellite peaks taken from both IR and Rama spectra against the phase angle, and **c** Raman spectra of the same samples. As a reference, IR and Raman spectra of PTFE are presented in (**d**, **f**) with a calculated dispersion curve [31] of PTFE in (**e**)

Figure 2a, c, respectively, presents IR and Raman spectra of perfluoro-n-alkanes having different chain lengths (C12–C16). The main peak of the ν_s_CF_2_ band in the IR spectra (Fig. 2a) appears at about 1155 cm^−1^ accompanying small many satellite peaks, and the positions of the satellite ones respond to the chain length [30]. Similar things are found for the Raman spectra (Fig. 2c): the main peak of the same band appears at about 734 cm^−1^ having satellite peaks. Since the C–F bond is characterized by a large permanent dipole moment and a small molecular polarizability [11, 12], the satellite peaks strongly appear in the IR spectra while they appear weakly in the Raman spectra as found in the inset.

The large difference of the main peak positions of the IR and Raman spectra (1155 and 734 cm^−1^, respectively) is explained by the selection rules for the coupled oscillator of the (CF_2_)_*m*_ chains [28, 32]. Except the perfluoroalkyl (*R*_f_)-specific ‘helical’ structure about the molecular axis [15, 16, 19], the molecular skeleton of the (CF_2_)_*m*_ chains basically is driven by the all-trans zigzag conformation that is also found in ordered hydrocarbons.

In the case of the (C**H**_2_)_*m*_ chains having the point group of *D*_2h_, they have the symmetric CH_2_ stretching vibration (ν_s_CH_2_) modes active for IR spectroscopy when the phase difference of the adjacent CH_2_ groups, *ϕ*, is π solely; whereas the Raman band is active for *ϕ* = 0 only [33]. Here, coupled oscillations having the rest phases are all inactive, which makes the IR and Raman bands of ν_s_CH_2_ mode very simple accompanying no satellite peaks. On the other hand, the coupled oscillations of the CH_2_ wagging vibrations (ωCH_2_) on an ordered alkyl chain are all active for both IR and Raman spectroscopy irrespective of the phases, and the peaks except *ϕ* = 0 and π thus appear as small many satellite peaks, which is known as BP [34–37]. The progression peaks of the ωCH_2_ mode has already been studied in detail, and the correlation between each peak position and its phase was theorized by the dispersion curve (Fig. 2e) [35, 36, 38–41]. In other words, this curve is the function connecting the positions of *ϕ* = 0 and π (Fig. 2d, f).

Now, let us get back to the R_f_-involved compounds. The ν_s_CF_2_ mode also gives many satellite peaks because of the helical conformation [16, 42]. Actual analysis of R_f_ compounds in terms of BP is made for the polymer of polytetrafluoroethylene (PTFE) (Fig. 2b) as shown by the dispersion curve in Fig. 2e, [43] and some limited oligomer compounds mostly for oligomers having an even number chain length [29]. Since we employ the series of compounds having both even and odd numbers, we have checked the satellite peaks in Fig. 2a are truly attributed to the BP peaks of the ν_s_CF_2_ mode. If they are all attributed to BP, a very similar dispersion curve to that of PTFE should be figured out by plotting the satellite peaks.

Therefore, the satellite peaks appeared in the IR and Raman spectra of perfluoro-n-alkanes (C12–C16) in Fig. 2a, c, respectively, are plotted against *ϕ* following the analytical procedure presented by Rabolt and Fanconi [42]. The red and blue circles are taken from the IR and Rama spectra, respectively. The phase, *ϕ*, was calculated using Eq. (1) where *N* is the number of repeating units. [29, 42]

1 $$\phi = \frac{k\pi }{{N + 1}}\;{\text{where}}\;k = 1,2,3, \ldots ,N.$$

The plot is figured out to have a dispersion curve in the wavenumber range between 1155 and 734 cm^−1^ (Fig. 2b), which reproduces the curve for PTFE (Fig. 2e). In this manner, the satellite peaks of the oligomer compounds with various chain lengths have all proved to be attributed to BP of the ν_s_CF_2_ mode. The confirmed BP peaks will then be employed for analysis of conformational change of R_f_ groups.

### Analysis of band progression peaks

**Fig. 3:**
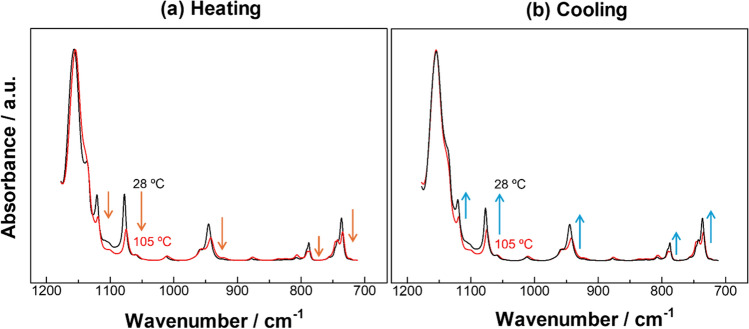
IR spectra of n-C_15_F_32_ at the beginning and the end of the temperature changes in the **a** heating and **b** cooling processes between 28 and 105 ºC

Figure 3 presents temperature dependent IR spectra in the BP region of one of the powder samples of perfluoroalkane, n-C_15_F_32_. The sample was once heated from ambient temperature, *T*_min_ = 28 ºC, up to *T*_max_ = 105 ºC (Fig. 3a), and it was soon cooled back to the ambient temperature (Fig. 3b).

In Fig. 3a, only the two spectra at the start and end temperatures are selected and presented for better visibility. As found in Fig. S1, all the BP peaks of ν_s_CF_2_ are monotonously decreased in absorbance on heating. This reminds us of a similar thing found for the BP peaks of the ωCH_2_ mode observed for the n-alkyl derivatives: the satellite peaks decrease on heating and disappear when attaining the melting point (Table 1) [34, 44–47] .

**Table 1:** Comparison of conformational changes between hydrocarbons and R_f_ oligomers. rCH_2_ stands for the CH_2_ rocking vibration. Other notations are given in the text

	Compounds	Chemical bond	Normal modes giving BP	Conformational change in terms of point group
Hydrocarbons	Saturated fatty acids	C–H	ωCH_2_, rCH_2_	*D*_2h_ → *C*_2v_
R_f_ oligomers	Perfluoroalkanes (C12 or longer)	C–F	ν_s_CF_2_	*D*_15_ → *D*_2h_

The disappearance of the BP peaks while the main peak stays almost unchanged has already been established to be attributed to the conformational change from *D*_2h_ to *C*_2v_ in the case of hydrocarbon chains as described “the breaking of the all-trans chain structure” [47]. In a similar manner, therefore, the decrease of the progression peaks of n-C_15_F_32_ keeping the main peak are also considered to reflect the conformational change of the R_f_ chain during the heating process (Table 1).

In fact, the temperature change of the heating process is mostly within Phase I of the phase diagram (Fig. 1), which corresponds to the molecular symmetry change from *D*_15_ toward *D*_2h_ (Table 1) [23, 48]. Since the untwisting change accompanies disorder (or defects) in the twist [17]; however, this change cannot be pursued by diffraction technique only. In this manner, the conformational change in Phase I has first been confirmed by IR spectroscopy through monitoring the BP peaks.

**Fig. 4:**
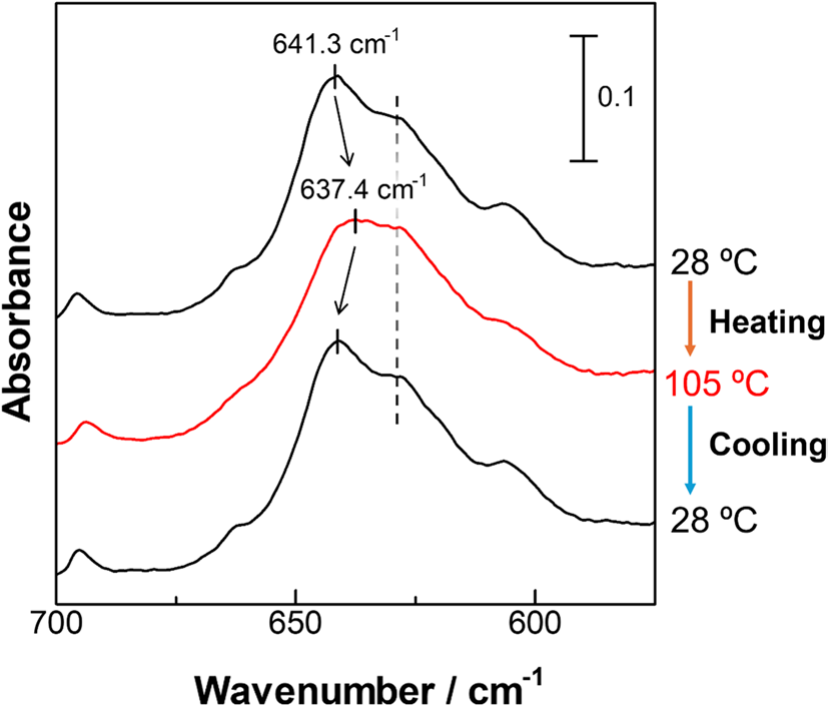
IR spectra of n-C_15_F_32_ thermally annealed in the range from 28 to 105 ºC

According to Zerbi and co-workers, the conformational change of PTFE from *D*_15_ to *D*_2h_ should give an IR band shift of an A_2_ mode from 640 to 625 cm^−1^ that is predicted by using the GF matrix method on many different conformation models [48]. The IR bands in a relatively low wavenumber region can be measured if diamond (lowest limit is 525 cm^−1^) is used for the ATR element, and the DTGS detector (lowest limit is 400 cm^−1^) is employed in place of the MCT detector (lowest limit is 650 cm^−1^). Representative IR spectra of n-C_15_F_32_ actually measured in the thermal annealing process between 28 and 105 °C are presented in Fig. 4. In the spectra of this oligomer compound, similar band positions are recognized at ca. 641 and 627 cm^−1^ that are both close to the calculated ones of PTFE. When the sample is heated, however, the band at 627 cm^−1^ is impervious to the heating, and only the band at 641 cm^−1^ is shifted to a lower wavenumber. Since the band at 641 cm^−1^ gets back to the original position when the temperature is cooled back, this is recognized as a reversal phenomenon that supports our analysis using the BP peaks. However, the disagreement from the calculated prediction indicates that analysis on these low wavenumber bands is difficult to discuss the conformational change solely, probably because band position is influenced not only by the conformational change, but also by the molecular distance between the adjacent molecules, i.e., crystal expansion.

**Fig. 5:**
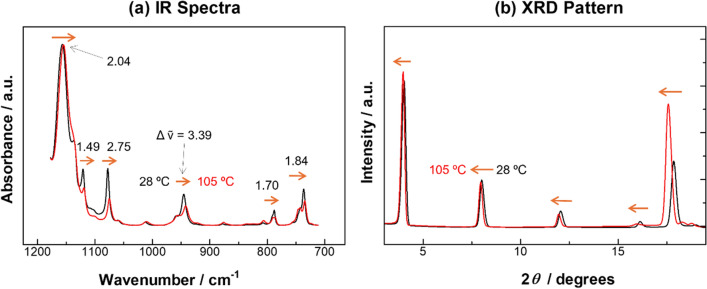
Influences of heating from 28 to 105 °C on **a** IR spectra and **b** XRD patterns of n-C_15_F_32_

In this sense, the BP peaks are revisited to inspect carefully in terms of peak positions. Fig. 5a shows peak shifts before and after the heating from 28 to 108 ºC. Since some of the peaks are overlapped with other bands, the shifts vary a little, but they are all shifted to the low wavenumber side. In general, a lower-wavenumber shift is commonly found for peaks relevant to both ν_s_CH_2_ and ν_s_CF_2_ modes, which is found when the molecular conformation is getting more ordered to be close to the conformation of *D*_2h_ [13, 49, 50]. In the case of R_f_ compounds, however, the conformation does not change almost at all indicating ignorable shift at an ambient temperature below 30°C [50]. Above the transition temperature of 30 °C (Phase I), on the other hand, a clear conformational change is expected, and molecular packing would also happen induced by the conformational change.

### Molecular packing analysis by XRD

The molecular packing was analyzed in the same temperature range by the XRD technique as found in Fig. 5b. All the peaks in the pattern show a lower shift of the diffraction angle, 2*θ*, with an increases of temperature, which means that all the interplanar distance, *d*, are getting larger as expected on considering Bragg’s law (Eq. (2)).

2 $$2d\sin \theta = n\lambda .$$

Here, *n* and *λ* are the diffraction order and the wavelength of the X-ray, respectively. The XRD pattern tells us that the molecular arrangement is basically kept unchanged, but the intermolecular distance is a little larger. As stated by Quarti and co-workers [26], the R_f_ chains are basically packed in a hexagonal manner at ambient pressure, and the intermolecular distances are a little changed to be pseudohexagonal in Phase I because of the defects in the chain, which agrees with our present results.

## Conclusion

Currently available phase diagram of R_f_ compounds is solely on PTFE revealed by XRD, neutron scattering, thermal expansion, and ultrasound waves. These techniques cooperatively work out for finding phase transitions, crystal structure and dispersion curves. This physical information is powerful to characterize each phase to reveal what is involved in the phase in qualitative manner. Nonetheless, quantitative analysis of the molecular symmetry having defects is expected especially for Phase I.

In the present study, we have confirmed that the oligomer perfluoro-n-alkanes covering both even and odd chain lengths yield clear BP peaks of the ν_s_CF_2_ mode in especially IR spectra that are all on the theoretical dispersion curve beautifully. The satellite peaks are all responsible to the temperature change in a reversible manner, which confirms that the change is driven by the molecular conformational (or symmetry) change, not by the crystallization process. Since the ν_s_CF_2_ main peak is nearly impervious to the temperature change, the BP peaks are highly useful for discussing the symmetry change in Phase I. In the future, studies on multivariate analysis of these peaks should be employed for quantitative discussion of the symmetry change. Besides, the BP and main peaks exhibit a reversal shift on the temperature change. Since the IR peak intensity measured by the ATR technique depends on the amount of the sample and the pressure applied on the sample during the measurement, the peak shift would be more useful for better quantitative discussion. Peak shifts are, however, not suitable for multivariate analysis and an analytical innovation is necessary for the quantitative shift analysis.

### Supplementary Information

Below is the link to the electronic supplementary material.

Supplementary file1 (DOCX 113 kb)

## Data Availability

The data that support the findings of this study are available from the corresponding author upon reasonable request.
