# Supplementary material for: Molecular symmetry change of perfluoro-n-alkanes in ‘Phase I’ monitored by infrared spectroscopy
Source: Anal Sci. 2024 Jun 14;40(9):1723–31. doi: 10.1007/s44211-024-00611-w (PMC11358247; doi:10.1007/s44211-024-00611-w)
Supplement: Supplementary file 1 — Supplementary file1 (DOCX 113 kb) [file 44211_2024_611_MOESM1_ESM.docx]

Molecular Symmetry Change of Perfluoro-n-alkanes in ‘Phase I’ Monitored by Infrared Spectroscopy

Taisuke Araki, Takayuki Oka, Nobutaka Shioya and Takeshi Hasegawa*

Laboratory of Chemistry for Functionalized Surfaces, Division of Environmental Chemistry, Institute for Chemical Research, Kyoto University, Gokasho, Uji, Kyoto 611-0011, Japan

**Supplementary Information**

*To whom correspondence should be addressed.

htakeshi@scl.kyoto-u.ac.jp


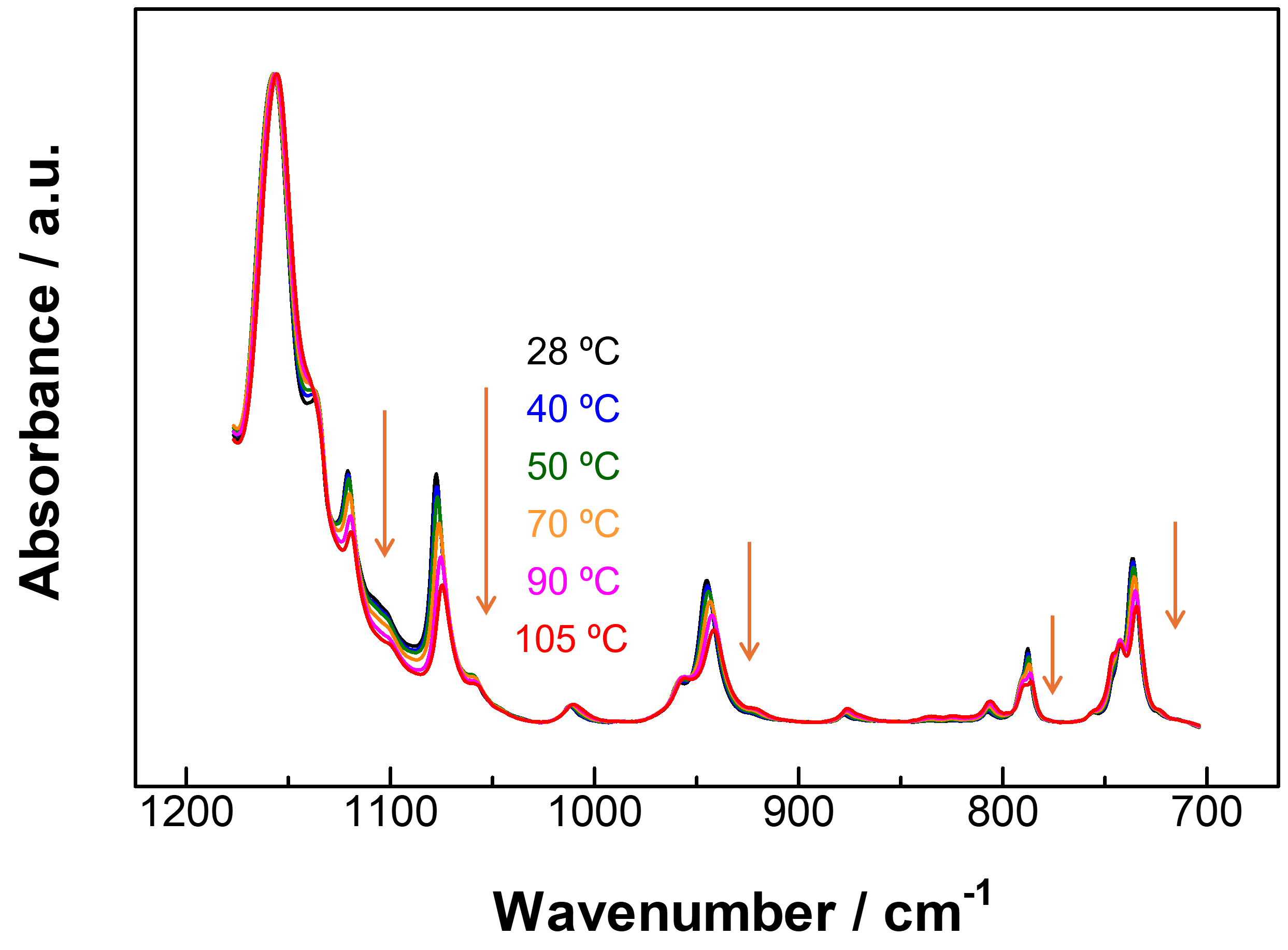


Figure S1. Six IR spectra of n-C_15_F_32_ in the heating process between 28 and 105ºC. The temperatures for the individual spectra are shown in the figure along an arrow.

**Supplementary Information:** Figure S1 presents temperature-dependent IR ATR spectra of the solid sample of n-C_15_F_32_ in the temperature range from *T*_min_ = 28ºC up to *T*_max_ = 105ºC. The experimental method and the conditions are the same as those for obtaining the spectra in Figure 3 except the number of accumulations. The accumulation was carried out 32 times for each spectrum.
